# EBF1 expressed in the dermal papilla regulates hair type and length

**DOI:** 10.1016/j.gendis.2024.101261

**Published:** 2024-03-07

**Authors:** Hongzhi Song, Leslie Lei Zhang, Wei-qi Zhong, Eve Qian Chen, Xi-Xi Qiu, Zi-Ling Tang, Xin-Hua Liao

**Affiliations:** a School of Medicine, Shanghai University, Shanghai 200444, China; b School of Life Sciences, Shanghai University, Shanghai 200444, China; c School of Environmental and Chemical Engineering, Shanghai University, Shanghai 200444, China

Loss of excessive hair growth causes psychological distress in many people, seriously affecting their social confidence and quality of life. The dermal papilla (DP) plays a pivotal role in regulating hair follicle (HF) morphogenesis and hair growth. However, the genes that govern the DP function in HF have not been extensively investigated. Genome-wide association studies have reported that two single-nucleotide polymorphisms (rs929626 and rs1081073) of the transcription factor EBF1 (early B-cell factor 1) are associated with male pattern baldness.^1^ Additionally, in *Ebf1* whole-body knockout mice, hair follicles fail to reenter the anagen phase after the first hair cycle, presumably because of the lack of intradermal adipocyte precursors.^2^ These findings suggested that EBF1 plays a vital role in hair loss-related diseases.

Here, we analyzed the chip data for gene expression levels in different populations of cells within the skin (Affymetrix array data of postnatal day 4 (P4) skin, accession number: GSE3142). Transcription factors were selected and ranked based on the expression fold-change in dermal papilla cells (DPCs) relative to other cell populations. We identified numerous transcription factors that were enriched in the DP (Table S1), and *Ebf1* ranked first among all the transcription factor genes. *Ebf1* was highly expressed in DPCs but was also expressed at lower levels in dermal fibroblasts, and almost no expression was observed in hair matrix cells, outer root sheath cells, and melanocytes (Fig. S1A). Additionally, the expression data of transcription factors in different cell populations in the skin obtained through RNA sequencing (RNA sequencing data of embryonic day 14.5 skin, accession number: GSE70288; RNA sequencing data of P5 skin, accession number: GSE77197) were also used to confirm that *Ebf1* was specifically enriched in DPCs (Fig. S1B, C), which is consistent with our analysis results for the chip data.

We introduced DP-specific *Cre* mice to target *Ebf1* within the DP conditionally and to study its function. We previously reported the enrichment of LEPR expression in DP.^3^ Observation of the skin sections of *Lepr-Cre* and *Rosa26-tdTomato* double-transgenic mice revealed that the reporter gene was expressed in the DP during the telogen phase (Fig. S2A). Our findings are consistent with those reported in 2022.^4^ We generated *Ebf1* conditional knockout (CKO) mice by crossing *Ebf1 fl/fl* with *Lepr-Cre*. Homozygous *Ebf1* CKO (*Lepr-Cre*, *Ebf1 fl*/*fl*) and CTR (*Ebf1 fl*/*fl*) mice were confirmed by genotyping (Fig. S2B). The homozygous CKO mice were as normal and healthy as their CTR littermates. There was no detectable change in hair color. The specific knockout of *Ebf1* in DPCs was verified by performing quantitative PCR on DPCs from both groups, which were sorted by flow cytometry using LEPR antibodies^3^ (Fig. S2C).

To investigate the effect of EBF1 deficiency on the hair cycle, we first shaved the back hair at P20 (the first telogen phase) and observed the hair regrowth over time (Fig. S3A). The back skin turns black when entering the anagen phase owing to the accumulation of pigments in the HF. We could only visually observe that the skin darkening of CKO mice was marginally slower than that of CTR mice in the first anagen phase. This difference was not evident in the graphs (Fig. S3B). The hair growth cycle is not well synchronized after the second telogen phase. Therefore, we used the percentage of the hair growth area of the total shaved area (hair growth score) as an indicator of the emergence of the second anagen phase. We shaved the back hair at P45 (the second telogen phase) and recorded the hair regrowth over time (Fig. 1A, B). Statistical analysis showed that CKO mice entered the second anagen phase significantly slower than CTR mice (Fig. 1C). We also used a repetitive depilation model to further investigate the effects of prolonged absence of EBF1 in DPCs on hair growth. At P50, we performed the first depilation on the mice and recorded the hair regeneration on days 14 and 21 (Fig. 1D). The next round of depilation was performed after hair regrowth. We found that mice with EBF1 deficiency in DPCs exhibited delayed hair regeneration on the seventh day after the second depilation. This delay became more apparent after three and four rounds of depilation. Statistical analysis of hair growth scores revealed significant differences between the CKO and CTR groups (Fig. 1E).

**Figure 1:**
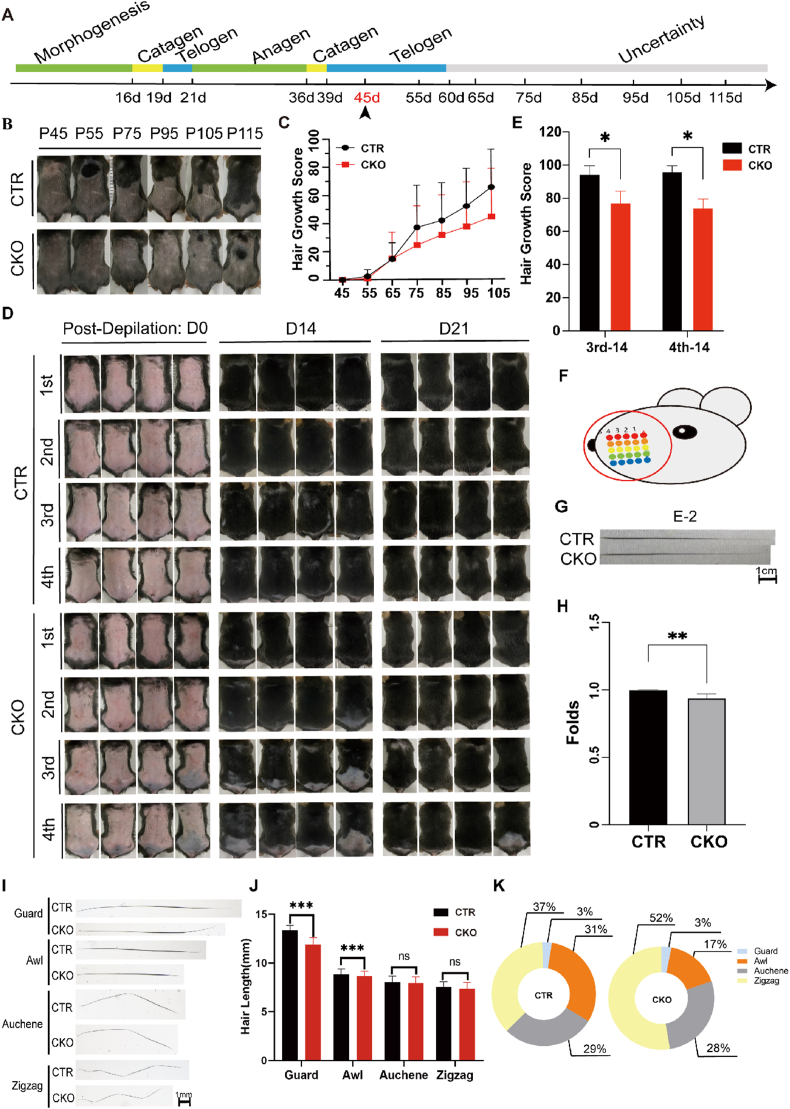
EBF1 expressed in the dermal papilla regulates hair type and length. **(A)** The diagram illustrating the timing of hair shaving during the telogen phase at P45 and showing the hair regrowth over time. **(B)** Representative images of hair regrowth of the CKO and the CTR mice after hair shaving at P45 over time. **(C)** Statistical analysis of hair growth scores over time in CTR and CKO mice (*n* = 7, male). **(D)** Four pairs of CKO and CTR mice underwent four rounds of depilation and were photographed on days 14 and 21 after depilation (*n* = 4, male). **(E)** Statistical analysis of hair growth scores on day 14 after the third and fourth depilation (*n* = 4, male; ∗*P* < 0.05). **(F)** Schematic representation of the location of mouse whiskers (rows A–E and arcs 1–5). **(G)** Representative images of whiskers at the E-2 position from CKO and CTR mouse. **(H)** Statistical analysis of whisker length at 15 positions from CKO and CTR mice. The length of CTR whiskers was normalized as 1 (*n* = 3, male; ∗∗*P* < 0.01). **(I)** Representative images of four different types of hair plucked from the back skin of CTR and CKO mice. **(J)** Statistical analysis of the length of four different types of hair in CTR and CKO mice, with 20 hairs for each hair type from each mouse (*n* = 4, male). **(K)** Comparison of proportions of four different hair types between the CTR and CKO mice (*n* = 20, male; ∗∗∗*P* < 0.001; ns, *P* > 0.05).

We examined the structure of mouse HF using hematoxylin-eosin staining and the proliferation status of HF epithelial cells using Ki67 immunostaining and found no changes after deletion of *Ebf1* in DPCs (Fig. S4A, B). This may be because EBF1 has a weak effect on the HF growth, making it difficult to detect changes in these semi-quantitative experiments. To further investigate the impact of *Ebf1* depletion on hair growth, we compared the length of whiskers in pairs, which is related to hair growth and can be measured quantitatively. Statistical analysis revealed that in mice with loss of EBF1 in DPCs, the length of whiskers was marginally shorter than that in the control group (Fig. 1F–H).

DPCs play a crucial role in hair type determination.^5^ To investigate the influence of EBF1 on hair type, we plucked hair at the same positions on the backs of non-shaved mice at P45 and classified them as Guard, Awl, Auchene, and Zigzag hairs (Fig. 1I). Compared with the CTR group, the CKO group had slightly shorter hair lengths for all four hair types (Fig. 1J), which was consistent with the results for whisker length results (Fig. 1H). Additionally, we calculated the proportion of all plucked hair for each hair type. In the CTR group, Awl and Zigzag accounted for 31% and 37% of the total proportion, respectively, whereas in the CKO group, they accounted for 17% and 52% of the total proportion, respectively (Fig. 1K). The proportions of Guard and Auchene hairs did not change significantly. This indicates that the loss of EBF1 in DPCs significantly decreased the proportion of Awl hairs and increased the proportion of Zigzag hairs.

In summary, we found that EBF1 is highly enriched in DPCs and that the ablation of *Ebf1* in DPCs in mice specifically delays the onset of the anagen phase of hair growth, resulting in shorter hair length and altered proportions of different hair types. Our study sheds light on the functions of two male pattern baldness-risk single-nucleotide polymorphisms located intronically within *EBF1* and offers a potential target for the prevention and treatment of hair loss-related diseases.

## Ethics declaration

All experiments involving animals were approved by the Experimental Animal Ethics Committee of Shanghai University.

## Author contributions

HZS: methodology, investigation, and data curation. LZ: methodology, investigation, and writing-original draft. WQZ: methodology, investigation, and data curation. EQC: methodology, data curation, and investigation. XXQ and ZLT: validation. XHL: conceptualization, methodology, supervision, writing-review & editing, and funding acquisition.

## Conflict of interests

The authors declared no conflict of interests.

## Funding

This work was supported by the 10.13039/501100012166National Key Research and Development Program of China (No. 2020YFA01130002) and the 10.13039/501100001809National Natural Science Foundation of China (No. 81972563).
