# Supplementary material for: EBF1 expressed in the dermal papilla regulates hair type and length
Source: Genes Dis. 2024 Mar 7;12(1):101261. doi: 10.1016/j.gendis.2024.101261 (PMC11532220; doi:10.1016/j.gendis.2024.101261)
Supplement: Multimedia component 1 [file mmc1.docx]

**Supplementary data for**

**EBF1 expressed in the dermal papilla regulates hair type and length**

# Hongzhi Song^a,b,c,1^, Lei Zhang^b,1^, Wei-Qi Zhong^a,b,1^, Eve Qian Chen^b,1^, Xi-Xi Qiu^b^, Zi-Ling Tang^b^, Xin-Hua Liao^∗b^

# ^a^School of Medicine, Shanghai University, Shanghai, 200444, China

# ^b^School of Life Sciences, Shanghai University, Shanghai, 200444, China

# ^c^School of Environmental and Chemical Engineering, Shanghai University, Shanghai, 200444, China

1 These authors contributed equally.

# *Corresponding author:

**Xin-Hua Liao, PhD**

Email: [xinhualiao@foxmail.com](mailto:xinhualiao@foxmail.com)

**This file includes:**

Methods and Materials

Supplementary Table 1

Supplementary Figure S1 to S4

# Material and methods

# Animals

*Lepr-Cre* mice (008320, the Jackson Laboratory), *Ebf1 fl/fl* mice (NM-CKO- 00065, Shanghai Model Organisms Center, Inc), *Rosa26-tdTomato* (007914, the Jackson Laboratory), C57BL/6 mice, *Flp* mice, were housed under specific pathogen-free (SPF) conditions. All experiments involving animals were approved by the Experimental Animal Ethics Committee of Shanghai University and conducted in accordance with the ARRIVE guidelines (Animal Research: Reporting of *In Vivo* Experiments) and the National Institutes of Health Guide for the Care and Use of Laboratory Animals.

**Generation and breeding of *Ebf1* CKO mice**

To generate *Ebf1* CKO mice, we constructed the targeting knockout vector containing homology arms, Loxp sites and FRT-flanked PGK-Neo drug selection cassettes. After linearization, the vector was electroporated into embryonic stem cells (ESC). Three positive ESC clones with recombination were screened out by long-range PCR, expanded and injected into the mice blastocysts to obtain chimeric mice with a high percentage of germ-line transmission. The chimeric mice were crossed with C57BL/6 mice to get heterozygous mice. When the homozygous mice were obtained, they crossed with *Flp* mice to obtain F1 heterozygous mice with deletion of the Neo drug selection cassette. After that, the *Ebf1 fl/fl* were mated with *Lepr-Cre* mice to obtain the *Lepr-Cre*, *Ebf1 fl/fl* cKO mice.

Mice genotyping was performed according to the manufacturer’s manual (PCR kit, AD201-01, TransGen Biotech Co, Ltd, China). Primers in experiments used for genotyping are as follows: *Ebf1*-F, CCAACAGCGAAAAGACCAAT; *Ebf1*-R, ACACAGGCCTCAAGAAGTGG; *Lepr*-F1, ATCTCCAGTAATCATTCCCCACT; *Lepr*-F2, TCAATACCGGAGATCATGC; *Lepr*-R, TTTCTTCTTTCCAGAGTTCAGATG.

# Skin embedding, immunostaining and HE staining

The back skins of mice at different phases of the hair cycle were embedded in OCT compound (Sakura Finetek, Torrance, CA, USA). The sections were cut at a thickness of 6-9 µm, fixed in 4% paraformaldehyde (PFA), and immunostained with Ki67 antibody (AB15580, Abcam). HE staining was conducted according to a manufacturer’s protocol (G1121, Beijing Solarbio Science & Technology Co, Ltd.). Briefly, tissue blocks were sectioned and fixed in 4% paraformaldehyde (PFA). The sections were stained with Hematoxylin solution for several minutes and washed with tap water. They were incubated in Blue with Bluing solution for 10s-1min and washed with tap water. After being dyed with Eosin solution for 30s-2min and washed, they were dehydrated in alcohol and sealed with resinene.

# DPCs isolation, RNA extraction and q-PCR

Preparation of single-cell suspension and DPCs sorting using flow cytometry have been described before ^[3]^. Briefly, skin samples were harvested from mice at postnatal day 4 (P4). The skin tissues were digested into single cells by collagenase and trypsin. LEPR antibody (JA73-01, HuaBio, Hangzhou, China) was added into the single-cell suspension, followed by the FITC conjugated secondary antibody. Approximately 5 minutes before sorting, propidium iodide was added to label dead cells. DPCs sorting was then performed by MoFlo XDP flow cytometers (Beckman Coulter Life Sciences). Total RNA was extracted from the sorted DPCs using TRIzol (15596018, Thermo Fisher Scientific). Subsequently, 1µg of total RNA was reverse transcribed using the Transcriptor FirstStrand cDNA Synthesis Kit (14231324, Roche), following the manufacturer’s instructions. For qPCR, Hieff qPCR SYBR Green Master Mix (10102ES08, Yeason biotechnology, Shanghai Co, Ltd.) was used for detecting the gene expression. The fold change in gene expression between CKO and CTR mice samples was determined using the 2^−ΔΔCT^ method. The housekeeping gene GAPDH was utilized as an internal control to normalize CT values across samples. The qPCR primers of *Ebf1* used are GGCTCCCCAACCTTCCTCAATGG and GCTGGTGAGAAGGAGAAGATGCC.

# Quantification of hair regrowth

For the first anagen, we used the ratio of the intensity of darkness of the back skin at P30 versus P20 as an indicator of the emergence of the anagen. The back skin was shaved at P20 and imaged with fixed parameter settings both at P20 and P30 under the control of the ruler. The average gray value of the image of the shaved area is calculated using Image J software. For the second anagen, we used hair growth score (percentage of hair growth area in the total shaved area) as an indicator of the emergence of anagen. Back skin was shaved at P45 and imaged with fixed parameter settings every 10 days afterward under the control of the ruler. The area with hair coating and the total shaved area were calculated using Image J software.

# Data analysis

All statistical results in this experiment are presented as mean ± standard error of the mean. Statistical differences were evaluated using a two-tailed paired Student’s t-test (GraphPad Prism v8.0, GraphPad Software, San Diego, CA, USA). The difference was considered significant when *P*<0.05 (*), *P*<0.01 (**), and *P*<0.001 (***).

| ***Gene symbol*** | **Mx** | **ORS** | **DF** | **DP** | **MC** | **m** | **RANK** |
| --- | --- | --- | --- | --- | --- | --- | --- |
| ***Ebf1*** | **0.1** | **1** | **420.5** | **1190** | **1** | **467.3104161** | **1** |
| *Sox18* | 11 | 27 | 721 | 1634 | 13 | 40.00306663 | 2 |
| *Prrx1* | 5 | 14 | 994.5 | 1108 | 24 | 30.81848199 | 3 |
| *Zic1* | 12 | 13 | 263.5 | 1362 | 22 | 44.16671847 | 4 |
| *Hoxc8* | 86 | 706 | 363 | 1796 | 23 | 11.96938755 | 5 |
| *Trps1* | 427 | 160 | 360 | 2835 | 138 | 11.74547817 | 6 |
| *Cebpd* | 57 | 176 | 1101 | 1625 | 202 | 7.476904134 | 7 |
| *Plagl1* | 63 | 513 | 2202.5 | 3734 | 945 | 7.332007551 | 8 |
| *Pbx1* | 74 | 159 | 324.5 | 987 | 147 | 6.412493423 | 9 |
| *Cebpa* | 395 | 999 | 239.5 | 2080 | 46 | 8.100407212 | 10 |
| *Peg3* | 131 | 508 | 1466 | 982 | 81 | 3.293616188 | 11 |
| *Klf9* | 94 | 194 | 1617 | 990 | 428 | 2.953686183 | 12 |
| *Mitf* | 62 | 85 | 288 | 964 | 2972 | 3.719752608 | 13 |
| *Junb* | 1072 | 909 | 1473 | 1838 | 207 | 2.489502501 | 14 |
| *Nfix* | 632 | 3200 | 2154 | 1567 | 71 | 2.101242713 | 15 |
| *Egr1* | 2869 | 5583 | 4425.5 | 5438 | 526 | 2.200658415 | 16 |
| *Smad6* | 323 | 156 | 122.5 | 893 | 761 | 3.41106105 | 17 |
| *Cebpb* | 1647 | 1492 | 1240.5 | 1563 | 88 | 2.17181842 | 18 |
| *Zfp36l2* | 476 | 1892 | 750 | 1111 | 120 | 2.082195053 | 19 |
| *Cbfb* | 889 | 1211 | 986.5 | 2008 | 1048 | 1.954958936 | 20 |
| *Fos* | 3383 | 4881 | 5961.5 | 5648 | 1684 | 1.574046572 | 21 |
| *Id2* | 672 | 202 | 233.5 | 935 | 1236 | 2.101624445 | 22 |
| *Hbp1* | 466 | 573 | 662 | 960 | 717 | 1.608947273 | 23 |
| *Xbp1* | 678 | 824 | 1941.5 | 1320 | 986 | 1.298019232 | 24 |
| *Tsc22d1* | 1692 | 1565 | 122525.5 | 2891 | 2560 | 0.538521199 | 25 |
| *Son* | 1159 | 1546 | 1521.5 | 1899 | 1076 | 1.451044316 | 26 |
| *Id1* | 1742 | 703 | 604.5 | 853 | 149 | 1.480139058 | 27 |
| *Adnp* | 827 | 1058 | 1191.5 | 1349 | 998 | 1.335697178 | 28 |
| *Id4* | 339 | 1656 | 988 | 1260 | 1521 | 1.314722204 | 29 |
| *Id3* | 2586 | 886 | 1401 | 1713 | 1078 | 1.255965119 | 30 |

**Table S1** Transcription Factors Enriched in DP

Mx: Matrix, ORS: Outer Root Sheath, DF: Dermal Fibroblast, DP: Dermal Papilla. MC: Melanocyte. *M* = *DP/Mx*, *O* = *DP/ORS* , *D* = *DP/DF*, *C* = *DP/MC.*$m=∜MODC$*.*

**Supplementary Figures**

**
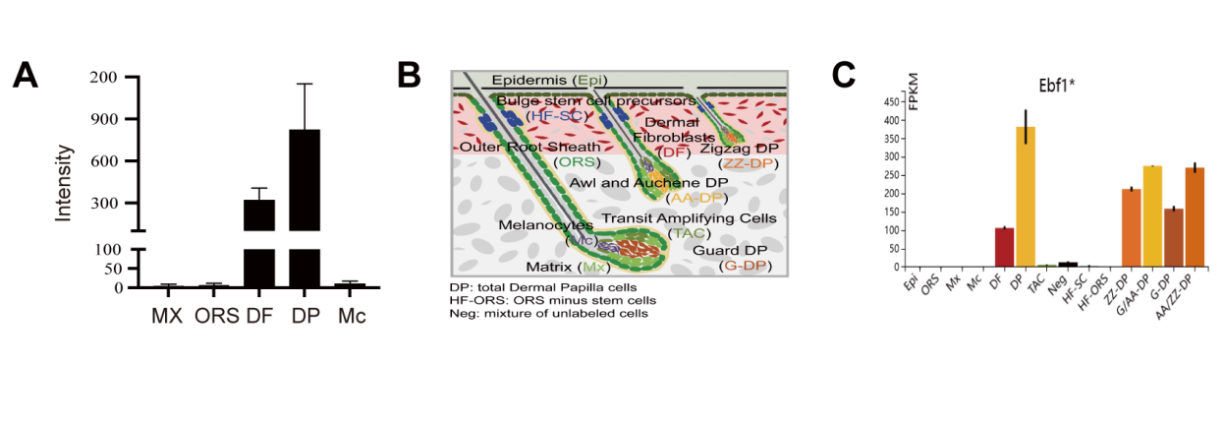
**

**Figure S1** Data analysis reveals that *Ebf1* is highly enriched in DPCs. **(A)** Expression of *Ebf1* mRNA in all cell populations of the skin at postnatal day 4 (P4) based on DNA microarray data. **(B)** Schematic representation of back skin at P5. **(C)** Expression of *Ebf1* mRNA in all cell populations of the skin at P4 based on RNA-seq data.

**
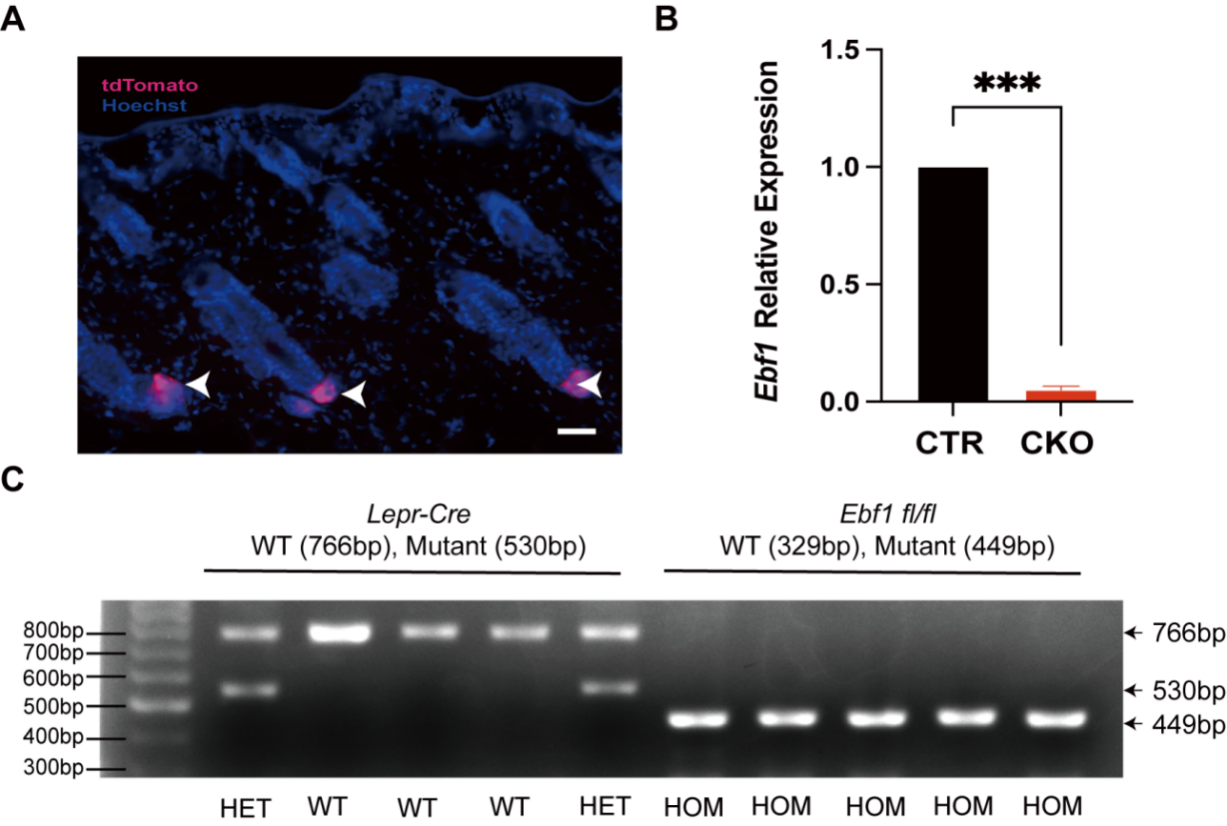
**

**Figure S2** Generation and genotyping of *Lepr-Cre*, *Ebf1 fl*/*fl* conditional knockout mice. **(A)** Immuno-fluorescence of skin section from *Lepr-Cre* and *Rosa26-tdTomato* double-transgenic mice at P50 telogen phase. Hoechst (blue: nuclear marker), tdTomato (red: reporter). The white arrows point to DPCs. **(B)** Genotyping of conditional knockout mice. Lane 1: Marker; Lanes 2-6: Gel electrophoresis analysis for identification of *Lepr-Cre* mice; Lanes 7-11: Gel electrophoresis for identification of *Ebf1 fl*/*fl* mice. WT, wild-type; HET, heterozygote; HOM, homozygote. **(C)** The expression of *Ebf1* in DPCs in *Lepr-Cre*, *Ebf1 fl*/*fl* male mice. q-PCR was used to evaluate the *Ebf1* relative expression at P4. Scale bar, 30 µm (n=4, ****P<*0.001).

**
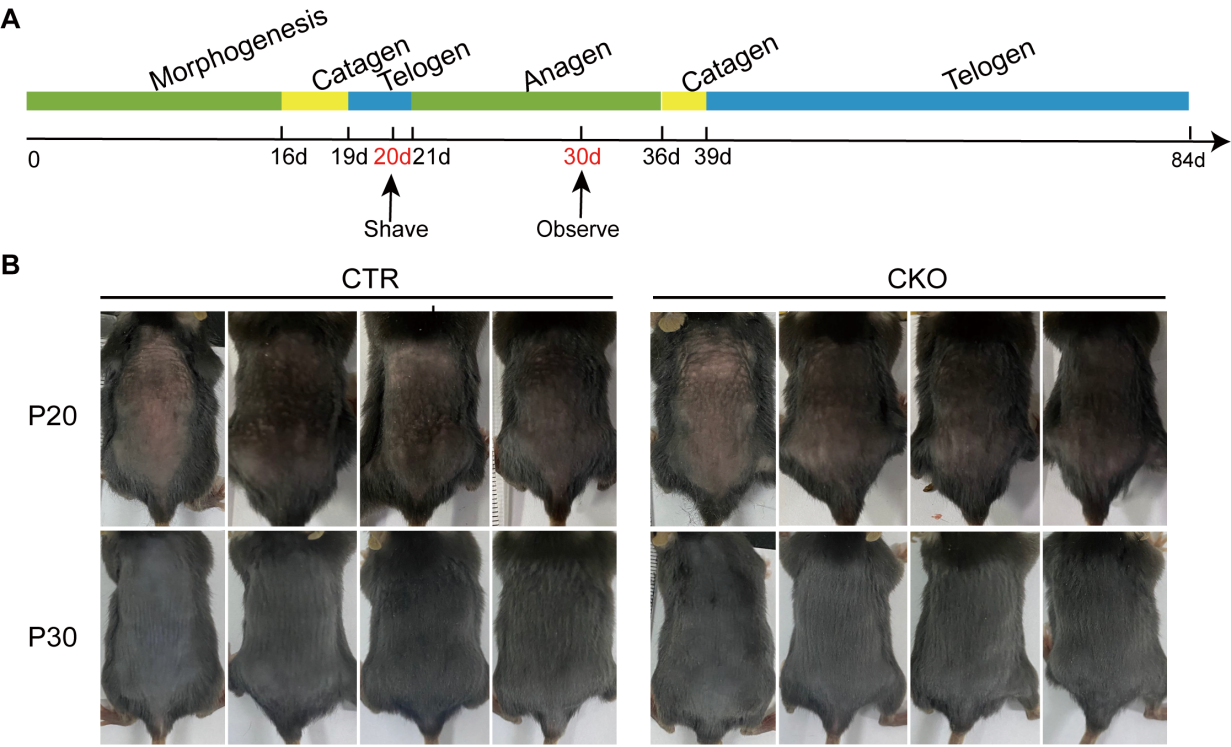
**

**Figure S3** Loss of EBF1 in DPCs leads to a delay of the onset of anagen. **(A)** Diagram depicting the timing of hair shaving at P20 during the telogen phase and photographic observations at P30 during the anagen phase. **(B)** Photographic recordings of the back skin of CTR and CKO mice at P20 and P30 (n=4, male).

**
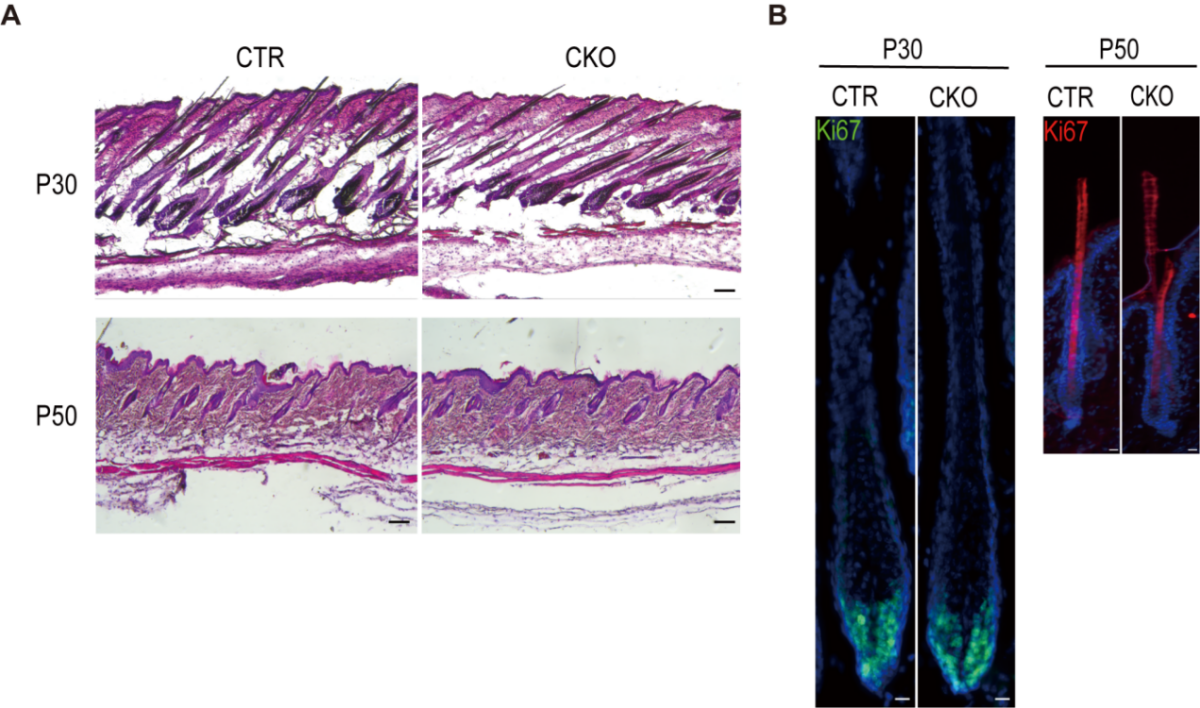
**

**Figure S4** Loss of EBF1 in DPCs did not have detectable effects on the structure of HF and proliferation of HF cells. **(A)** H&E staining of back skin sections from CKO and CTR mice at P30 and P50. Scale bar: 100 µm. **(B)** Immunostaining of Ki67 (proliferation marker) in the back skin sections from CKO and CTR mice at P30 and P50. Scale bar: 20 µm.
